# Editorial: Mobile Elements and Plant Genome Evolution, Comparative Analyzes and Computational Tools

**DOI:** 10.3389/fpls.2021.735134

**Published:** 2021-09-24

**Authors:** Ruslan Kalendar, Francois Sabot, Fernando Rodriguez, Gennady I. Karlov, Lucia Natali, Karine Alix

**Affiliations:** ^1^HiLIFE Institute of Biotechnology, University of Helsinki, Helsinki, Finland; ^2^National Laboratory Astana, Nazarbayev University, Nur-Sultan, Kazakhstan; ^3^DIADE, University of Montpellier, CIRAD, IRD, Montpellier, France; ^4^Josephine Bay Paul Center for Comparative Molecular Biology and Evolution, Marine Biological Laboratory (MBL), Woods Hole, MA, United States; ^5^Laboratory of Applied Genomics and Crop Breeding, All-Russia Research Institute of Agricultural Biotechnology, Moscow, Russia; ^6^Department of Agriculture, Food and Environment, University of Pisa, Pisa, Italy; ^7^GQE – Le Moulon, Université Paris-Saclay, INRAE, CNRS, AgroParisTech, Gif-sur-Yvette, France

**Keywords:** plant genome evolution, transposable elements (TE), retrotransposon (RTN), retrotransposon (RTN)-based markers, transposon display (TD)

## Transposable Elements Turn Out to be Functional

Multiple changes that occur constantly in the plant genome allow an organism to develop from a single-celled embryo to a multicellular organism. A significant part of these changes is associated with the recombination activity of numerous classes of interspersed repeats. These numerous families of interspersed repeats were often called “junk DNA” as first, they were not associated with any vital protein-coding processes. Now, more and more clues indicate that such repeated DNA might play major roles in the genome as functional “non-coding” DNA (Pennisi, 2012; Ariel and Manavella, 2021). Transposable elements (TEs), such as DNA transposons and retrotransposons, are the main part of these interspersed repeats (Vitte et al., 2014). The diverse families of retrotransposons are highly abundant genetic elements that are related to retroviruses (Wicker et al., 2007). Although retrotransposons are not “true” mobile elements like DNA transposons—from the comparison of their transposition mechanisms, retrotransposable elements (RTEs) are notably constitutive of heterochromatin and form a variety of major chromosomal structures such as centromeres (Bennetzen and Wang, 2014) and represent the main intergenic part of the plant genome (Kalendar et al., 2020). RTE mobility is ensured through an RNA intermediate, allowing a Copy-And-Paste approach for their transposition. Their own-encoded RNA is reverse transcribed using their own (or not) encoded enzymes, that will re-create from the single-strand RNA matrix a double-strand DNA at a new location. This reverse transcription can be either through extra-chromosomal DNA within a nucleocapsid (e.g., and implies RT/RNAseH + INT enzymes for LTR retrotransposons) or directly at the insertion site (e.g., Target-Prime Reverse transcription mechanism, with only the RT as a minimal set of enzymes for LINEs and SINEs; Wicker et al., 2007). Such Copy-And-Paste mechanisms allow a quick invasion of naive genomes, and is responsible for massive genome increase in a few periods of time (Piegu et al., 2006). Such invasions belong to a few number of initial active copies, called Master copies; however, while these copies must be transcriptionally active (i.e., able to produce RNA), they can be translationally inactive and may rely on other copies for enzymatic activities (Sabot and Schulman, 2006). When recently inserted, neo-copies can be related phylogenetically because of their sequence identity; subsequent mutations (point mutation, recombination, and so on) will then occur and their lineage can then be complex to recompose. Once inserted, each copy has its own evolutionary history and some may acquire biological role (or some part of the copy) and being excepted by their host. Some RTEs can provide evolutionary advantages to the host and were demonstrated to play a significant role in plant adaptation (Song and Cao, 2017). If the fact that TEs can be beneficial to the host is now accepted, recent advances in the field has placed RTEs at the center of the current debate on eukaryotic and notably plant evolution. To advance this important research field, in the Research Topic “Mobile Elements and Plant Genome Evolution, Comparative Analyses, and Computational Tools” we focused on the efficiency of new genomic tools for the discovery of TEs, and highlighted some recent studies on the role of mobile elements in the evolution of the host genome, as well as on genome-wide comparative analysis and profiling of transposable elements.

## Mobile Element and Host Genome Evolution

Different retrotransposon families, each with its own lineage and structure, may have been active at distinct phases in the evolution of a species. Retrotransposon sequences bear the promoters that bind the nuclear factors of transcription initialization and initiate RNA synthesis by polymerases II or III. In the article entitled “Additional ORFs in Plant LTR-Retrotransposons” by Vicient and Casacuberta, LTR-retrotransposons that carry additional but not retrotransposon-specific open reading frames (aORF) were discovered and analyzed. This discovery reinforces the unique potential of LTR-retrotransposons as evolutionary tools, thanks to their ability to provide new genetic variants within a genome. The presence of antisense aORFs in characterized LTR-retrotransposon families from various angiosperm species indicates a putative role of these coding regions in the transposition process. To clarify the functions of these aORFs, further analysis of TE transcript splicing variants and thus access to the TE functional diversity would be very valuable.

## Transposable Elements as Drivers of Structural and Functional Variations

Thanks to their remarkable diversity in size, genomic organization and transposition process, TEs are important drivers of genome evolution and species diversity (Vicient and Casacuberta, 2017). Keidar-Friedman et al. and Bariah et al. reported the potential impact of miniature transposable element insertions on the expression of genes in different wheat species, with the articles entitled “The Evolutionary Dynamics of a Novel Miniature Transposable Element in the Wheat Genome” and “Where the Wild Things Are: Transposable Elements as Drivers of Structural and Functional Variations in the Wheat Genome.” TE-mediated genomic rearrangements and insertions of mobile genetic elements in gene-rich regions may act as mutagens and contribute to genome alteration (Vicient and Casacuberta, 2017). TE-mediated epigenetic modifications lead to phenotypic diversity, genetic variation, and environmental stress tolerance. Potential TEs also contribute to genome plasticity and have a dramatic impact on the genetic diversity and evolution of the wheat genome. Using transposon display (Kalendar et al., 2021) and genome-wide profiling analysis of insertional polymorphisms of transposable elements (Kalendar and Schulman, 2006), the authors discovered large genomic rearrangement events, such as deletions and introgressions in the wheat genome. High-throughput bioinformatics with next-generation sequencing (NGS) were key tools in these studies (Vondrak et al., 2020). Structural rearrangements, gene duplications, and variation in TE contents may have a large impact on the overall genomic structure and may be responsible for phenotypic diversity (Belyayev et al., 2010). In the article entitled “Genome Size Variation and Comparative Genomics Reveal Intraspecific Diversity in *Brassica rapa*,” Boutte et al. investigated structural variants and changes in the repetitive fraction between two accessions of *Brassica rapa*, and characterized genome-size variation among a core collection, using comparative genomics and cytogenetic approaches. Detection of large genomic variants between different cultigroups of the same species *B. rapa* highlighted the potential impact of the differential insertion dynamics of repeated elements in the intraspecific variability of genome size and chromosomal structure. This study also justifies the recent and general effort to construct pangenomes when access to the genomic diversity of a given species is sought.

TEs contribute also to the driving force in the evolution of epigenetic regulation and have a long-term impact on genomic instability and evolution. Remnants of RTEs appear to be overrepresented in transcription regulatory modules and other regions conserved among distantly related species, which may have implications for our understanding of their impact on speciation. In the article entitled “Sequencing Multiple Cotton Genomes Reveals Complex Structures and Lays Foundation for Breeding,” Pan et al. revealed that post-polyploidization of cotton genome instability resulted in numerous genomic structural changes that were accompanied by the expansion of LTR-retrotransposon families with an unbalanced contribution from the constituent genomes of cotton, with TEs from the D-genome being more active than TEs from the A-genome. This study also demonstrated that TE transposition activity coincided with cotton genome divergence, supporting the major role of TEs in genome shaping and speciation. Finally, the authors depicted the evolutionary past of cotton plants, which were recursively affected by polyploidization, like all plant lineages (Alix et al., 2017): a decaploidization contributed to the formation of the genus *Gossypium* prior to the well-known neo-tetraploidization that accompanied the formation of the currently widely cultivated cotton species. The resulting functional innovation comes directly from the diversity of duplicated genes, as suggested by their uncommonly high evolutionary rates.

## Chromosome Evolution With Transposable Elements and Satellite DNAs

The centromere is the unique part of the chromosome that combines a highly conserved function with a large variability of its constituent DNA sequences. In the article entitled “Functional *Allium fistulosum* centromeres comprise arrays of a long satellite repeat, insertions of retrotransposons and chloroplast DNA” Kirov et al. studied the functional centromere organization in the large-sized chromosomes of the species *Allium fistulosum* and *A. cepa*. They demonstrate that long and high-copy repeats are associated with insertions of retrotransposons and chloroplast DNA sequences, with tandem repeats being also constitutive of the centromeres. This article provides an insight into the major role of repetitive DNA in centromere function. Among evolutionary factors, repetitive sequences play multiple roles notably in sex chromosome evolution. As such, the *Spinacia* genus serves as an ideal model to investigate the evolutionary mechanisms underlying the transition from homomorphic to heteromorphic sex chromosomes. This was studied in the article entitled “Genome-Wide Analysis of Transposable Elements and Satellite DNAs in *Spinacia* Species to Shed Light on Their Roles in Sex Chromosome Evolution” by Li et al. Major repetitive sequence classes were identified in male and female genomes of *Spinacia* species and their ancestral relative sugar beet in order to decipher the evolutionary processes of sex chromosome evolution using NGS data. The differences of repetitive DNA sequences correlate with the formation of sex chromosomes and satellite DNAs are highly accumulated at the sex determination locus, demonstrating the role of repetitive DNA in sex chromosome evolution.

## Author Contributions

RK, FS, and KA prepared the draft. All authors listed have made a substantial, direct and intellectual contribution to the work, and approved it for publication.

## Funding

This work was supported by the Science Committee of the Ministry of Education and Science of the Republic of Kazakhstan in the framework of program funding for research (AP08855353).

## Conflict of Interest

The authors declare that the research was conducted in the absence of any commercial or financial relationships that could be construed as a potential conflict of interest.

## Publisher's Note

All claims expressed in this article are solely those of the authors and do not necessarily represent those of their affiliated organizations, or those of the publisher, the editors and the reviewers. Any product that may be evaluated in this article, or claim that may be made by its manufacturer, is not guaranteed or endorsed by the publisher.

## References

[B1] Alix K. Gerard P. R. Schwarzacher T. Heslop-Harrison J. S. P. (2017). Polyploidy and interspecific hybridization: partners for adaptation, speciation and evolution in plants. Ann. Bot. 120, 183–194. 10.1093/aob/mcx079 28854567 PMC5737848

[B2] Ariel F. D. Manavella P. A. (2021). When junk DNA turns functional: transposon-derived non-coding RNAs in plants. J. Exp. Bot. 72, 4132–4143. 10.1093/jxb/erab073 33606874

[B3] Belyayev A. Kalendar R. Brodsky L. Nevo E. Schulman A. H. Raskina O. (2010). Transposable elements in a marginal plant population: temporal fluctuations provide new insights into genome evolution of wild diploid wheat. Mobile DNA 1:6. 10.1186/1759-8753-1-6 20226076 PMC2836003

[B4] Bennetzen J. L. Wang H. (2014). The contributions of transposable elements to the structure, function, and evolution of plant genomes. Annu. Rev. Plant Biol. 65, 505–530. 10.1146/annurev-arplant-050213-035811 24579996

[B5] Kalendar R. Raskina O. Belyayev A. Schulman A. H. (2020). Long tandem arrays of cassandra retroelements and their role in genome dynamics in plants. Int. J. Mol. Sci. 21:2931. 10.3390/ijms21082931 32331257 PMC7215508

[B6] Kalendar R. Schulman A. (2006). IRAP and REMAP for retrotransposon-based genotyping and fingerprinting. Nat. Protocols 1, 2478–2484. 10.1038/nprot.2006.377 17406494

[B7] Kalendar R. Shustov A. Schulman A. (2021). Palindromic sequence-targeted (PST) PCR, version 2: an advanced method for high-throughput targeted gene characterization and transposon display. Front. Plant Sci. 12:691940. 10.3389/fpls.2021.691940 34239528 PMC8258406

[B8] Pennisi E. (2012). Genomics. ENCODE project writes eulogy for junk DNA. Science 337, 1159–1161. 10.1126/science.337.6099.1159 22955811

[B9] Piegu B. Guyot R. Picault N. Roulin A. Sanyal A. Kim H.. (2006). Doubling genome size without polyploidization: dynamics of retrotransposition-driven genomic expansions in *Oryza australiensis*, a wild relative of rice. Genome Res. 16, 1262–1269. 10.1101/gr.5290206 16963705 PMC1581435

[B10] Sabot F. Schulman A. H. (2006). Parasitism and the retrotransposon life cycle in plants: a hitchhiker's guide to the genome. Heredity 97, 381–388. 10.1038/sj.hdy.6800903 16985508

[B11] Song X. Cao X. (2017). Transposon-mediated epigenetic regulation contributes to phenotypic diversity and environmental adaptation in rice. Curr. Opin. Plant Biol. 36, 111–118. 10.1016/j.pbi.2017.02.004 28273484

[B12] Vicient C. M. Casacuberta J. M. (2017). Impact of transposable elements on polyploid plant genomes. Ann. Bot. 120, 195–207. 10.1093/aob/mcx078 28854566 PMC5737689

[B13] Vitte C. Fustier M. A. Alix K. Tenaillon M. I. (2014). The bright side of transposons in crop evolution. Brief Funct. Genom. 13, 276–295. 10.1093/bfgp/elu002 24681749

[B14] Vondrak T. Avila Robledillo L. Novak P. Koblizkova A. Neumann P. Macas J. (2020). Characterization of repeat arrays in ultra-long nanopore reads reveals frequent origin of satellite DNA from retrotransposon-derived tandem repeats. Plant J. 101, 484–500. 10.1111/tpj.14546 31559657 PMC7004042

[B15] Wicker T. Sabot F. Hua-Van A. Bennetzen J. L. Capy P. Chalhoub B.. (2007). A unified classification system for eukaryotic transposable elements. Nat. Rev. Genet. 8, 973–982. 10.1038/nrg2165 17984973

